# The SMOS-Derived Soil Water EXtent and equivalent layer thickness facilitate determination of soil water resources

**DOI:** 10.1038/s41598-020-75475-x

**Published:** 2020-10-27

**Authors:** Boguslaw Usowicz, Mateusz Lukowski, Jerzy Lipiec

**Affiliations:** Institute of Agrophysics, Polish Academy of Sciences, Doświadczalna 4, 20-290 Lublin, Poland

**Keywords:** Environmental sciences, Hydrology

## Abstract

The assessment of water resources in soil is important in understanding the water cycle in the natural environment and the processes of water exchange between the soil and the atmosphere. The main objective of the study was to assess water resources (in 2010–2013) in the topsoil from satellite (SMOS) and in situ (ground) measurements using the *SWEX_PD* approach (Soil Water EXtent at Penetration Depth). The *SWEX_PD* is a result of multiplying soil moisture (SM) and radiation penetration depth (*PD*) for each pixel derived from the SMOS satellite. The *PD*, being a manifold of the wavelength *λ*_0_ equal to 21 cm, was determined from the weekly SMOS L2 measurement data based on the real and imaginary part of complex dielectric constant. The *SWEX_PD* data were compared with soil water resources (*WR*) calculated from the sum of components derived from multiplication of soil moisture (*SM*) and layer thickness in nine agrometeorological stations located along the eastern border of Poland. Each study site consisted of seven neighbouring Discrete Global Grid pixels (nodes spaced at 15 km) including the central ones with agrometeorological stations. The study area included different types of soils and land covers. The agreement between the water resources obtained from the *SWEX_PD* and ground measurements (*WR*) was quantified using classical statistics and Bland–Altman's plots. Calibrated Layer Thickness (CLT = *d*_*bias*_) from 8 to 28 cm was obtained with a low values of *bias* (close to zero), limits of agreements, and confidence intervals for all the *SWEX_PD*, depending on the pixel location. The results revealed that the use of the *SWEX_PD* for assessing soil water resources is the most reliable approach in the study area. Additionally, the data from Bland–Altman plots and the equation proposed in these studies allowed calculation of the Equivalent Layer Thickness (ELT = $d_{ei}^{SWEX}$), which corresponds to the water resources derived from the SMOS satellite at the same time as (*SM*) measurements performed in the agrometeorological stations. The ranges of the mean, standard deviation, minimum, maximum, and coefficient of variation (CV) of ELT among all pixels and stations were 8.28–28.7 cm, 3.27–12.66 cm, 3.03–10.87 cm, 19.23–94.97 cm, and 24.72–98.79%, respectively. The ranges of the characteristics depended on environmental conditions and their means were close to the values of the calibrated layer thickness. The impacts of soil texture, organic matter, vegetation, and their interactive effects on the differentiation and agreement of soil water resources obtained from *SWEX_PD* vs. data from ground measurements in the study area are discussed. Further studies are required to address the impact of the environmental factors to improve the assessment of soil water resources based on satellite SM products (retrievals).

## Introduction

Soil water resources play a significant role in the agriculture and the entire environment^1–4^. They influence soil-atmosphere relations through exchange of energy, fluxes of water and greenhouse gases, and latent heat flux during evaporation^5–8^. Consequently, they are an important variable for weather predicting and climate projection^9–11^, including forecasting extreme events^12^. Monitoring the soil moisture and water resources is necessary for numerous applications such as agricultural drought assessment, irrigation scheduling, soil and crop management^13,14^, and ground water recharge^15^.

Soil water resources data can be acquired from ground-based measurements or globally, using satellite techniques^16,17^. Satellite remote sensing facilitates investigation of large-scale areas where field observations are insufficient to provide data. Different frequencies including X, C, and L bands are used in space-borne satellites to estimate soil moisture content^18–20^. C and X bands have been used in some satellite sensors, e.g. AMSR-E and ASCAT, to determine surface (skin) soil wetness, whereas L-band (microwave) radiometers help to analyse near-surface (0–5 cm) soil moisture. Research indicates that the use of the L–band (21 cm) (1.4–1.427 GHz) is the most promising radiometry approach for estimation of soil moisture due to its higher sensitivity to the dielectric properties of soil and lower sensitivity to the vegetation layer^11,19,21^. Since 2009, this band has been used in the Soil Moisture and Ocean Salinity (SMOS) mission for delivering the brightness temperature and global mapping of near-surface (0–5 cm) soil moisture at a temporal resolution of 2 to 3 days^22,23^. In addition, this frequency is within the protected band for radio astronomy and exhibits minimum radio-frequency interference (RFI)^24^.

Microwave remote sensing of topsoil moisture data has been useful for many Earth systems including agriculture, weather forecasting, and determination of landslide potential and ground trafficability^19^. The usefulness and applicability of remotely sensed topsoil *SM* has been increased by new developments allowing assessment of water available for plants in the root-zone based on the topsoil moisture at a regional^25,26^ and global scale^27^. Reviews of relevant research literature indicate that the potential for the use of satellite global soil moisture products for many societal applications can be further enhanced by refining or developing adaptative scaling, data assimilation, and modelling schemes^19,22^.

An important parameter of satellite remote sensing is the radiation penetration depth (synonymously used with “sensing depth”), which corresponds to the depth derived from the satellite and is defined as a depth at which the intensity of electromagnetic waves decreases (is attenuated) by a factor of 1/*e* times^28^ (about 37%), where *e* is Euler's number equal to approximately 2.718. The penetration capability of microwave signals decreases with the increasing dielectric constant of the soil due to the increasing moisture content^29^. Furthermore, the Penetration Depth (*PD*) can be influenced by soil properties and land cover through their water content and attenuation at the L-band^19,30^. Besides, the *PD* decreases as the incident angle of the sensors increases^29^. Due to these interferences (relations), the retrievals of soil moisture and temperature profile information from diverse satellites can be comparable after precise determination of the penetration depths^11,31,32^.

*Validation* and *calibration* of space-borne observations by comparison with ground measurements are crucial in evaluating the quality of satellite products^17^. Therefore, in situ soil moisture monitoring systems have been established to calibrate and validate (Cal/Val) soil moisture data or brightness temperature (TB)^17,33,34^. An important factor leading to minimization of errors of satellite *SM* retrievals is precise matching the in situ measurement and the satellite sensing depth^11^. Frequently, sensors are placed at depths fitting to mathematical numerical simulations of soil moisture and soil temperature^11,35^ and/or result from the geometry of existing sensors that dictates the location depth^31^.

Although remote sensing using L-band provides valuable information, scientific improvements are still needed in terms of assessment of soil water resources^11,16,18,19^. Therefore, the aim of this study was to assess soil water resources using the new concept Soil Water EXtent at Penetration Depth (*SWEX_PD*) as the product of soil moisture (*SM*) and radiation penetration depth (*PD*), both from SMOS L2 satellite data. The *SWEX_PD*-SMOS-derived soil water resources were validated using ground-monitored soil moisture data at different depths in agrometeorological stations in Poland. Bland–Altman plots were used to quantify the differences between the soil water resources determined by *SWEX_PD*-SMOS and ground measurements. Assuming that the average water resources from these two methods are equal and their dispersion is within the strictly defined range of the limit of agreement (LoA) and confidence interval (CI), an approach has been proposed to determine the Calibrated Layer Thickness (CLT) and the Equivalent Layer Thickness (ELT) of soil water derived from the SMOS satellite.

## Materials and methods

### Study area

**Figure 1:**
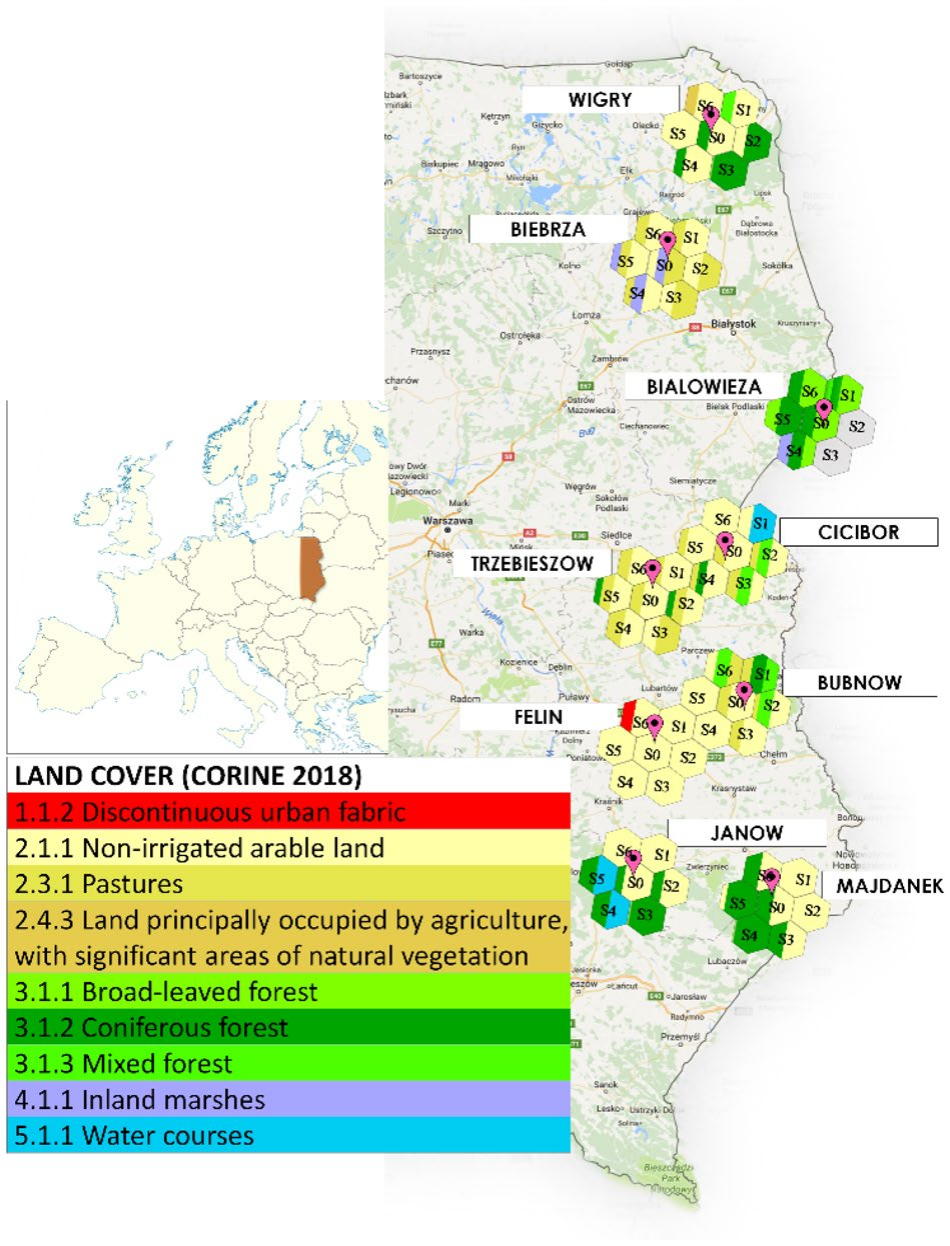
Agrometeorological stations and sites located along the eastern border of Poland with marked pixels from the SMOS satellite and with the structure of the land cover. The surface area occupied by the individual colours in the pixels roughly reflects the surface area occupied by the land cover symbolized by the respective colours. Background maps from Google Maps (https://www.google.com/maps/@52.44051119.441469,7z?hl = en), accessed 12 Apr. 2017. The background maps were modified using Microsoft Office PowerPoint 2016.

The study was conducted in 9 sites in the eastern part of Poland (Fig. 1). Each site is equipped with agrometeorological station measuring soil moisture (*SM*). The soil moisture data for 2010–2013 (obtained every 3 days) used in the study came from the SMOS satellite (SMUDP2 v. 551) and were provided over the ISEA-4H9 (icosahedral snyder equal area Earth fixed) grid referred to as the discrete global grid (DGG)^36^. The nodes were equally spaced at 15 km. Next, 7 DGG pixels per each site were chosen in a way that the central one (named S0) contained the agrometeorological station and the other 6 (named S1, S2, S3, S4, S5, S6) were bordering around. Real and imaginary part of complex dielectric constant data from SMOS were used to determine the penetration depth using the approach described below.

### Penetration depth

The information about soil near-surface layer water resources is required for scientific elucidation of the global water cycle. The idea presented in this paper was to create *SWEX_PD* (Soil Water EXtent at Penetration Depth) for representing the amount of sub-surface soil water, substituting the surface Soil Moisture (*SM*) given by SMOS SMUDP2 data files. The multiplying soil moisture (*SM*) and radiation penetration depth (*PD*) was first proposed by W. Marczewski to determine uncalibrated soil moisture resources from SMOS measurements^37^.

The Penetration Depth (*PD*) is derived from dielectric constants based on the Kirchhoff Approach (KA)^28^. The approach assumes that SMOS gathering Temperature Brightness (TB) of emitted radiation over the soil layer thickness *PD* facilitates not only polarimetric retrieval for *SM* but also estimation of *PD* with KA for incoherent radiation. TB is used for determination of the intensity of radiation, while the next aspect is its attenuation by the layer *PD*—a concept analogical to the optical thickness of soil (also determined by the water). It is believed that, in this way, the surface Soil Moisture may be coupled to the absolute measures of water mass under observation, in kilograms per a particular surface, and the volume of the layer on the *PD* thickness.

According to the Beer-Lambert law^28^, the intensity of an electromagnetic wave in a uniform media falls off exponentially along the propagation path as

1 $$I\left( z \right) = I_{0} e^{ - \alpha z}$$

where *I*_0_ is the electromagnetic wave in the air, *α* is the attenuation constant. We define the Penetration Depth (*PD*) for a path of *z* as a depth at which the intensity of electromagnetic waves decreases *e* times, where the Euler number *e* ≈ 2.718 defines 1 Neper in attenuation terms. We arbitrarily define:

2 $$PD = \frac{1}{\alpha }$$

At depth *z* = *PD*, the electromagnetic wave components (E, H) have 1/*e* of their initial value. Since the power of the *α* wave in a particular medium is proportional to the square of intensity, the power at Penetration Depth is 1/*e*^2^, which is 13.5% of its initial value. The Kirchhoff Approach requires equality of the attenuation for radiation waves propagated by emission from media in two directions, i.e. for outsourcing and incoming waves. The attenuation constant for an electromagnetic wave at a normal incidence angle is proportional to the imaginary part of the material’s refractive index *n*. It may be also expressed by the wave-extinction index *κ*:

3 $$\alpha = \frac{\omega }{c}{\text{Im}} \left( {n\left( \omega \right)} \right) = \frac{2\pi \kappa }{{\lambda_{0} }}$$

where *ω* is the angular frequency of radiation, *λ*_0_ is the wavelength, and *c* is the speed of light in vacuum. The complex refractive index *n* and complex dielectric constant *ε* equally represent the propagation and loss properties of the medium and (in non-ferromagnetic materials) are related by^28^:

4 $$\varepsilon = \varepsilon_{1} + i\varepsilon_{2} = \left( {n + i\kappa } \right)^{2}$$

where *ε*_1_ is the real part and *ε*_2_ is the imaginary part of the dielectric constant and *i* is the imaginary unit. After some simple transformations, one can obtain the conversions:

5 $$n = \sqrt {\frac{{\sqrt {\varepsilon_{1}^{2} + \varepsilon_{2}^{2} } + \varepsilon_{1} }}{2}} \; {\text{and}} \;\kappa = \sqrt {\frac{{\sqrt {\varepsilon_{1}^{2} + \varepsilon_{2}^{2} } - \varepsilon_{1} }}{2}}$$

and find the following formula for Penetration Depth:

6 $$PD = \left( {2\pi \sqrt {\frac{{\sqrt {\left( {Dielect\_Const\_RE} \right)^{2} + \left( {Dielect\_Const\_IM} \right)^{2} } - Dielect\_Const\_RE }}{2}} } \right)^{ - 1}$$

where *Dielect_Const* is the complex dielectric constant from SMOS L2 data, and *RE* and *IM* denote the real and imaginary parts of this complex number, respectively. *PD* is expressed in *λ*_0_, which for SMOS is 21 cm.

### Soil Water EXtent at Penetration Depth (SWEX_PD)

The *SWEX_PD* was proposed for representing the topsoil water amount in relation to the Soil Moisture (*SM*) at a depth corresponding to the radiometric Penetration Depth (*PD*) observed by SMOS in SMUDP2. The *SWEX_PD* concept couples surface SMOS data with the hydrology of deeper soil layers and may be an answer to the need of information about water retention in terms of water mass (in tons per square kilometre).

Uncalibrated “water resources in soil” in the time *j* step derived from the SMOS satellites were calculated using the *SWEX_PD*_*jk*_ as the product of soil moisture (*SM*_*jk*_) and radiation penetration depth (*PD*_*jk*_) for each SMOS pixel (*k* = 0, 1, 2,…*n*):

7 $$SWEX\_PD_{jk} = SM_{jk} \times PD_{jk}$$

${\text{SWEX}}_{\text{j}}={\text{SM}}_{\text{j}}\times {\text{PD}}_{\text{j}}$ ${\text{SWEX}}_{\text{j}}={\text{SM}}_{\text{j}}\times {\text{PD}}_{\text{j}}$ Therefore, *SWEX_PD*_*jk*_ is proportional to the soil moisture derived from SMOS but only in *PD*_*jk*_ (not applicable to water resources in hydrological terms). *PD*_*jk*_ was expressed in a wavelength *λ*_0_ equal to 21 cm and was determined on the basis of weekly SMOS L2 measurement data based on the dependence of the real and imaginary parts of the dielectric constant.

### Ground soil water resources (WR)

Ground soil moisture measurement data from various locations and depths were used to determine soil water resources (*WR*) with the time *j* step. *WR*_*j*0_ were obtained from the multiplication of ground measured soil moisture (*SM*_*i*0_) and layer thickness *d*_*i*_, *i* = 1, 2,…*m* in the central pixel (S0) and then compared with six neighbouring pixels (S1, S2, S3, S4, S5, S6). In the agrometeorological stations located along the eastern border of Poland (Fig. 1), four configurations of three sensors types were used to measure soil moisture. The sensors were installed on grassed soil in four configurations: (1) the PR2 probe was used (at 10, 20, 30, 40, 60, and 100 cm depths) (Delta-T) in four stations (Wigry, Biebrza, Białowieża, Trzebieszów), (2) ThetaProbe ML2x sensors were used (5, 20, and 30 cm) (Delta-T) in three stations (Cicibór, Janów Lubelski, Majdanek), (3) a combination of the ThetaProbe ML2x (5 cm) and the PR2 probe (10, 20, 30, 40, 60, and 100 cm) were used in one station (Bubnów), (4) TDR sensors (5, 10, 20, and 40 cm) (EasyTest) were used both on grassed (vegetated) and bare (arable) soil in one station (Felin-Lublin). Soil moisture data (*SM*_*i*_) were averaged weekly for each depth separately. Soil water resources (*WR*_*j*0_) were calculated from the sum of components derived from multiplication of soil moisture (*SM*_*i*0_) and layer thickness (*d*_*i*_, *i* = 1, 2,…*m*) and converted to wavelength *λ*_0_ units:

8 $${\text{WR}}_{{\text{j0}}} \left( {\frac{{{\text{SM}}_{{\text{i0}}} \times {\text{d}}_{{\text{i}}} }}{{{\uplambda }_{0} }}} \right) = \mathop \sum \limits_{{{\text{i}} = 1}}^{{\text{m}}} {\text{SM}}_{{\text{i0}}} \times \frac{{{\text{d}}_{{\text{i}}} }}{21},\;\;\;j = {1},{ 2} \ldots ,k$$

where: *m*—number of layers with a known thickness, *λ*_0_—wavelength (21 cm) used by the SMOS satellite to measure soil moisture, *j*—time step (week).

### Statistical approach

The uncalibrated (unscaled) *SWEX_PD*_*jk*_ water resources derived from the SMOS satellite were compared with the water resources (*WR*_*j*0_) derived from ground measurements. To this end, Bland–Altman plots^38^ were adopted as shown below. It was hypothesized that water resources are equal when the average of the differences *SWEX_PD*_*jk*_ – *WR*_*j*0_, the so-called *bias*, are close to or equal to zero:

9 $$bias = \frac{1}{n}\mathop \sum \limits_{j = 1}^{n} \left( {SWEX\_PD_{jk} - WR_{j0} } \right) \cong 0,$$

and the dispersion of the differences is finite and lies within a well-defined scatter path in the limit of agreement (LoA) and confidence interval (CI), which were calculated from the equations as: LoA = *bias* ± 1.96 × *s* (*s*—standard deviation), CI for *bias*: CI = *bias* ± *t* × (*s*^2^ *n*^–1^)^0.5^, and CI for LoA: CI = LoA ± *t* × (3*s*^2^ *n*^–1^)^0.5^, where *n* is the number of data and *t* is the value of t distribution with *n*–1 degrees of freedom^39^. The difference between the *SWEX_PD*_*jk*_ and (*WR*_*j*0_) derived water resources was adjusted according to the equation:

10 $$SWEX\_PD_{jk} - WR_{j0} = a\left( {\frac{{SWEX\_PD_{jk} + WR_{j0} }}{2}} \right) + b$$

**Figure 2:**
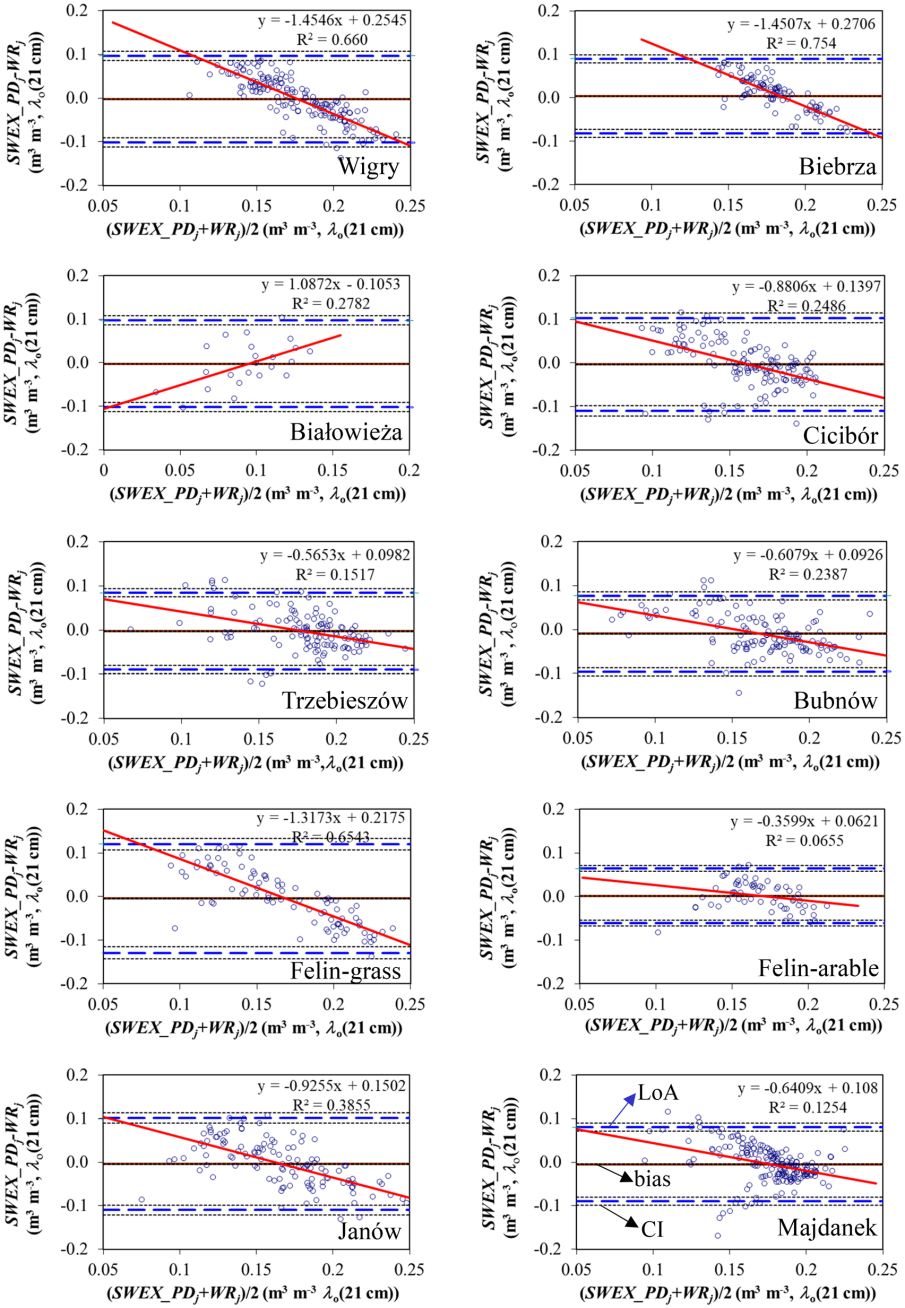
Bland–Altman plot *SWEX_PD*_*j*0_ − *WR*_*j*0_ vs. (*SWEX_PD*_*j*0_ + *WR*_*j*0_)/2 for S0 pixels depending on the location of the station. Explanations: *bias* line, limits of agreement (LoA), confidence intervals (CI) for the *bias* and LoA, regression lines (red) and equations with R^2^—determination coefficients.

Selection of the Calibrated Layer Thickness (CLT = *d*_*bias*_) at which the *bias* was close to zero or equal to zero was carried out. Depending on the type of sensors installed in the agrometeorological stations two steps coarse and accurate were used. The layer thicknesses in the coarse step for the first sensor in the configurations PR2 probe (1) and the ThetaProbe ML2x (2) were: *d*_*i*_ = 5, 10, and 15 cm and for other sensors they were *d*_*i*_ = 5 and 10 cm. For the configurations ThetaProbe ML2x (5 cm) and the PR2 probe (3) and TDR sensors (4) the layer thicknesses for the individual sensors were: *d*_*i*_ = 5 and 10 cm. The layer thickness of 1 cm was taken in the accurate step that allows to minimize the differences in soil water resources between those from satellite and ground-based measurements. The coarse step included the sum of the products of soil moisture (*SM*_*i*_) and the thickness of the layers given above, checking how much the *bias* differed from zero. When the *bias* was much less than zero, the values of *d*_*i*_ were decreased by the accurate steps (1 cm) and chosen so that the *bias* was close to zero. When the *bias* was greater than zero, *d*_*i*_ was increased by the coarse or accurate steps so that the *bias* was close to zero. LoA and CI were also calculated to estimate the spread of the analysed data, and the *a* and *b* parameters of the linear regression equation were determined (see example in Fig. 2, site Majdanek). Values obtained from the linear regression equation and the *bias* value (Eq. 11) at the intersection point are equal and correspond to the CLT.

11 $$SWEX\_PD_{jk} - WR_{j0} = a\left( {\frac{{SWEX\_PD_{jk} + WR_{j0} }}{2}} \right) + b = bias$$

Only points close to *bias* on the Bland–Altman plot correspond to the CLT and those much above or below *bias* have different layer thicknesses resulting in overestimated or underestimated water resources. To reduce the deviations from *bias* the layer thicknesses were calculated from the transformed regression equation (Eq. 12) so that the regression equation line overlap with *bias* line (Eq. 13). The layer thickness calculated in this way, referred to as ($d_{ei}^{SWEX}$), was named Equivalent Layer Thickness (ELT) that corresponds to water resources derived from the SMOS satellite.

12 $$SWEX\_PD_{jk}^{*} = \frac{{2\left( {bias - b} \right)}}{a} - WR_{j0}$$

13 $$d_{ei}^{SWEX} = \frac{{SWEX\_PD_{jk}^{*} \times d_{bias} }}{{SWEX\_PD_{jk} }} = \frac{{\left( {\frac{{2\left( {bias - b} \right)}}{a} - WR_{j0} } \right) \times d_{bias} }}{{SWEX\_PD_{jk} }}$$

where $SWEX\_PD_{jk}^{*}$ is the water resource at the intersection point of the regression equation and the *bias* lines.

### Ground measurements in agrometeorological stations

The ranges of sand, silt, and clay in the ground *SM* measurement stations were 22–97, 1–73, and 2–6% (Table 1). The Biebrza and Bubnów stations (sites) were partly covered by marsh soils containing up to 80% of organic matter. The amount of organic matter in some parts of the Białowieża site reached up to 52%. In the other stations, there was 1–3% of organic matter.

**Table 1:** Data on the study sites.

Name	Geographical location		Particle size distribution of the soil in the 0–30 cm layer (%)		
	Longitude (°)	Latitude (°)	Sand (2.0–0.05 mm)	Silt (0.05–0.002 mm)	Clay (< 0.002 mm)
Wigry	23.013536	54.060920	87	11	2
Biebrza	22.535407	53.301436	97	1	2
Białowieża	23.847912	52.707234	68	28	4
Cicibór	23.099275	52.069369	58	37	5
Trzebieszów	22.565413	51.987345	72	26	2
Bubnów	23.280621	51.374580	83	15	2
Felin	22.625077	51.219703	26	68	6
Janów	22.418174	50.691230	86	9	5
Majdanek	23.470604	50.478213	22	73	5

## Results

### Comparison of soil water resources from SWEX_PD with ground measurements WR

**Figure 3:**
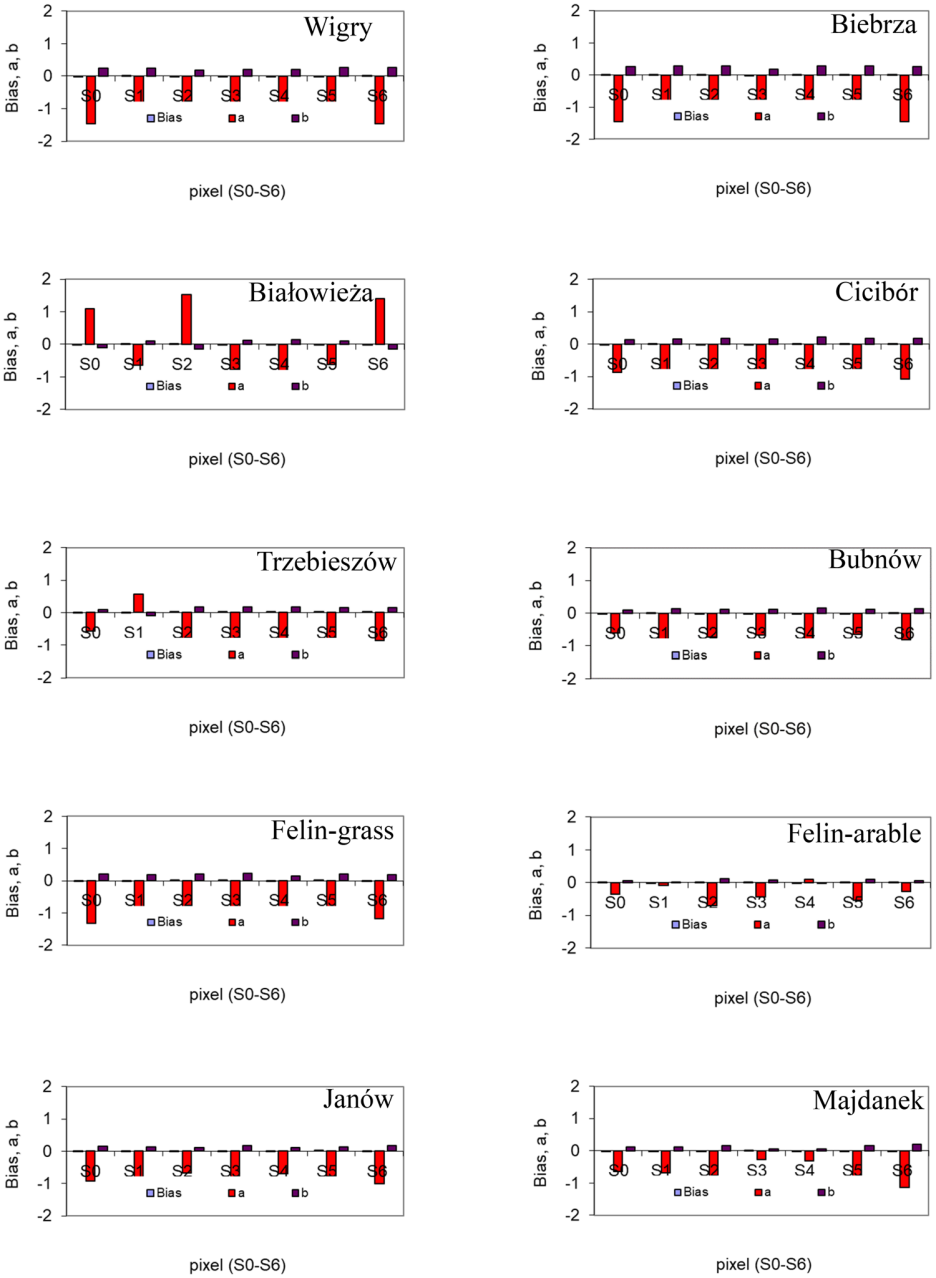
*Bias* and parameters of regression lines: a and b (y = ax + b) for S0–S6 pixels depending on the location of the site from the Bland–Altman plots.

The use of both coarse and exact steps depending on the *SM* sensor types and their installation depths allowed estimation (with an accuracy of 1 cm) of soil depths at which the soil water resources from ground measurements (*WR*_*j*0_) were similar or equal to those from the *SWEX_PD*_*jk*_ based on the satellite data, as quantified by the Bland–Altman plots. The statistics including the differences *SWEX_PD*_*jk*_ – *WR*_*j*0_, i.e. *bias*, LoA, CI, and the linear regression equation *SWEX_PD*_*jk*_ – *WR*_*j*0_ vs. (*SWEX_PD*_*jk*_ + *WR*_*j*0_)/2 for pixel S0 are shown in the Bland–Altman plots (Fig. 2). The linear regression equation parameters (*a*, *b*) for all pixels (S0-S6) are given in Fig. 3 along with *biases* at which the (*WR*_*j*0_) values were similar or equal to those from the *SWEX_PD*_*jk*_. The majority of *SWEX_PD*_*jk*_ − *WR*_*j*0_ vs. (*SWEX_PD*_*jk*_ + *WR*_*j*0_)/2 values were within the defined LoA, some were within CI, and only few were beyond the areas of both LoAs and CIs. Such an arrangement of the data and the *bias* being close to zero indicated that the effect of the random factor was not significant and that the Bland–Altman approach can be used for the statistical analysis.

As can be seen in Fig. 2, the LoA values for pixels S0 with *SM* sensors were generally in the range ± 0.1. Similarly, the LoAs for the other pixels (S1-S6) in each site were within this range (data not shown). It is worth noting that the largest and smallest LoAs were calculated for the grassed (> ± 0.1) and arable areas (around ± 0.07), respectively, in the same pixel Felin S0 (Fig. 2). Similarly, the CI values for *bias* and agreement limits (LoAs) were larger in the Felin-grassed than Felin-arable soil. As the line of equality (zero difference) lies between the confidence interval limits of *bias*, the latter is not significant. However, the CI of LoAs reached values around 0.02 in the Felin-grassed soil. This indicates that the estimated water resources from these two methods are more compatible in the Felin-arable soil. As can be seen in Fig. 3, the *bias* values are small (close to zero), positive or negative, in all sites and pixels. The linear regression coefficients (*a*) of the relationship *SWEX_PD*_*jk*_ – *WR*_*j*0_ vs. (*SWEX_PD*_*jk*_ + *WR*_*j*0_)/2 (in Bland–Altman plots) were mostly negative with respective values < 0.5, 0.5–1.0, and > 1 for 6, 33, and 26 cases. They had positive values (from 0.096 to 1.536) only in 3 pixels in the Białowieża site (S0, S2, S6) and 1 pixel both in Trzebieszów (S1) and Felin-arable (S4).

**Figure 4:**
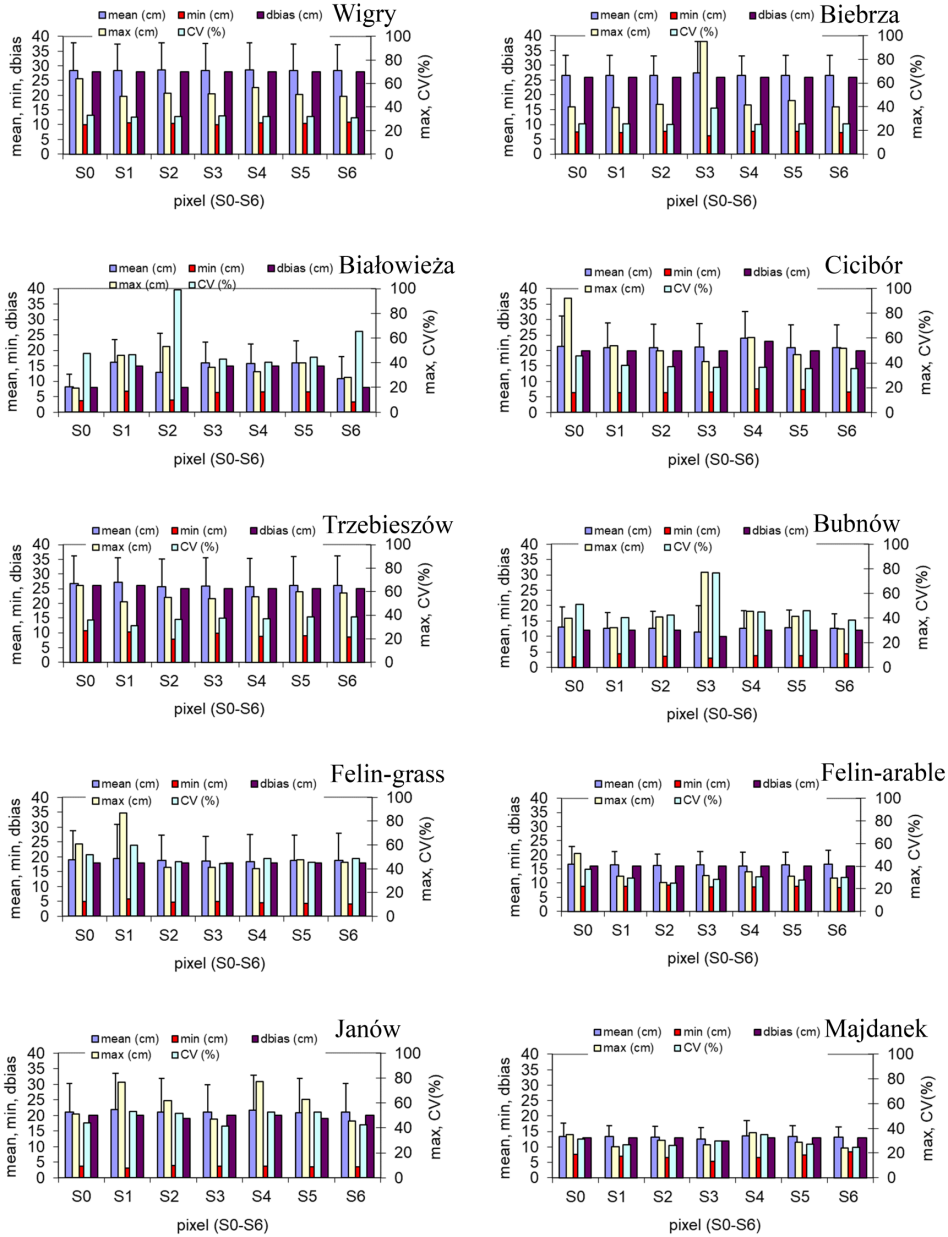
Statistical parameters of equivalent layer thickness (ELT) and calibrated layer thickness (CLT) for data from all pixels (S0–S6) in each station. Explanations: ELT—mean (from 8.28 to 28.7 cm) with bars of standard deviation (from 3.27 to 12.66 cm), minimum (from 3.03 to 10.87 cm), maximum (from 19.23 to 94.97 cm), and coefficient of variation CV(%) (from 24.72 to 98.79%), and CLT—dbias (from the surface up to 8–28 cm depth).

As shown in Fig. 4, the largest calibrated layer thickness derived from the SMOS (*d*_*bias*_) satellite was found in all or most pixels belonging to the Wigry, Biebrza, and Trzebieszów stations (from 26 to 28 cm), and lower values were observed in Majdanek, Felin-arable, and Bubnów (from 12 to 14 cm). The larger calibrated layer thickness indicates that the same amount of water in a given soil is distributed over a larger depth. The smallest and the largest differentiation of the calibrated layer thickness (from 8 to 15 cm) between the pixels was found in the Białowieża site with various land covers. These differences may implicate different drought responses depending on rooting and vegetation size.

### Equivalent water thickness

Based on both the results of the Bland–Altman approach and the equation (Eq. 13) proposed in this study, the equivalent layer thickness (ELT = $d_{ei}^{SWEX}$) was determined. The statistics of the ELT including the mean, SD, minimum, maximum, and CV for the central and neighbouring pixels in the 4-year study period (2010–2013) are given in Fig. 4. The overall ranges of variations of the mean ELT, standard deviation (indicated by bars), minimum, maximum, and coefficient of variation (CV) for all pixels and stations were 8.28–28.7 cm, 3.27–12.66 cm, 3.03–10.87 cm, 19.23–94.97 cm, and 24.72–98.79%, respectively. Based on the ELT values, the sites can be divided into three groups. The mean ELT (for all pixels) had the largest values in Trzebieszów, Biebrza, and Wigry (from 25.8 to 28.7 cm) and successively decreased in Felin-grassed, Janów, and Cicibór (from 18.4 to 23.9 cm) as well as Białowieża, Bubnów, and Majdanek Felin-arable (from 8.3 to 16.8 cm).

Overall, the ranges (differences between minimum and maximum values) of the mean, min., max. SD, CV, and *d*_*bias*_ values for (ELT) for all pixels and stations were 20.4 cm, 7.8 cm, 75.7 cm, 9.4 cm, 74.1%, and 20.0 cm, respectively (Table 2). The ranges of the maximum ELT values between the pixels (S0-S6) were the smallest in the Majdanek, Trzebieszów, and Wigry sites (to 20 cm); intermediate values were found in Felin (arable), Janów, Białowieża, Bubnów, and Felin (grass) (from 20 to 50 cm), and the largest values were determined in the Cicibór, Biebrza sites (above 50 cm). The largest CV ranges, i.e. 58.3 and 38.1%, recorded for Białowieża and Bubnów, respectively, were approximately 2.5–4.0 times larger than in the other stations (≤ 15.2%). The mean and min. values of the ELT in the particular stations were in 15 and 5 cases below 2 cm and above 2 cm, respectively.

**Table 2:** Ranges of the statistical parameters of equivalent layer thickness (ELT) and calibrated layer thickness (d*bias*) for data from all pixels in each station and from all pixels and stations.

Range, ELT (cm)	Wigry	Biebrza	Białowieża	Cicibór	Trzebieszów	Bubnów	Felin grass	Felin arable	Janów	Majdanek	All pixels and stations
Mean	0.30	0.90	7.83	3.07	1.39	1.66	0.96	0.68	0.91	1.15	20.4
Min	0.92	1.51	3.35	1.23	2.86	1.39	1.55	0.76	0.75	3.06	7.8
Max	15.1	55.7	34.1	51.6	14.0	46.2	47.0	25.5	31.6	12.6	75.7
SD	0.60	3.99	8.73	2.35	1.67	3.85	3.31	2.18	2.84	1.50	9.4
CV (%)	2.1	13.8	58.3	10.1	7.7	38.1	15.2	12.3	11.6	10.2	74.1
d*bias*	0.0	0.0	7.0	3.0	1.0	2.0	0.0	0.0	1.0	1.0	20.0

Explanations: *min* minimum, *max* maximum, *SD* standard deviation, and *CV (%)* coefficient of variation.

## Discussion

Penetration depth (satellite sensing depth) and resultant soil water resources at a particular time depend on the soil textural composition, which determines the water-holding capacity^19,30^. In general, the penetration depth increases and the water-holding capacity decreases successively in clay, silt, and sandy soils. In our study, the effect of soil texture on the calibrated layer thickness determined by *SEWX_PD* or soil water resources derived from the SMOS satellite was not explicit. For example, a positive effect of the sand fraction can be clearly seen by comparison of the calibrated layer thickness from 19 to 26 cm for agricultural soils containing 58–86% of sand (Cicibór, Trzebieszów and Janów sites) with that from 13 to 18 cm for soils containing 22–26% of sand (and 68–73% of silt) (Majdanek and Felin stations—Table 1, Fig. 4). However, a negative effect of sand was observed while comparing the lower calibrated layer thickness in Biebrza (26 cm) with that in Wigry (28 cm) with the respective sand content of 97 and 87%. The lower calibrated layer thickness "seen" in the Biebrza station may result from the presence of organic matter-rich marshes (up to 80%), which retain much more water than inherently permeable sandy soils^40^. Also in the Bubnów site, the "seen" calibrated layer thickness was relatively small (10–12 cm) despite the relatively high content of sand (83%). Bubnów, similarly to Biebrza and Białowieża (52%), also had large amounts of organic matter in soil reaching up to 80%.

Our results showed the largest ranges of the 4-year calibrated layer thickness (CLT = *d*_*bias*_) derived from the SMOS satellite (8–15 cm), equivalent layer thickness (ELT = $d_{ei}^{SWEX}$) (from 8.3 to 16.09 cm), and coefficient of variation (CV) (from 40.5 to 99%) (Fig. 4) between the pixels in the Białowieża site. This site is situated in Białowieża National Park on the territory of Poland and Belarus with well-preserved forests and biological diversity, including the rare European bison. The area within the station is highly heterogeneous due to the spatial variation of soil organic matter content, textural composition, wetness, and vegetation. The area offers a diversity of conditions that influence the SMOS penetration depth retrievals. The analysis of the satellite-based pictures has demonstrated that the differences in the "seen" soil water resources (similarly, the differences in the equivalent layer thickness) between the pixels can be influenced by the type of predominant trees, i.e. either deciduous or coniferous. This can be illustrated by comparison of the relatively high CLT of 15 cm in pixel S5 covered predominantly by a coniferous forest with that of 8 cm in pixels S0 and S6 with predominance of broad-leaved forests (Figs. 1 and 4). Broad-leaved trees vs. coniferous trees have a larger surface leaf area and (thus) water content, which may have a masking effect on the water "seen" in the soil^17^ due to the presence of hydrogen stored in the vegetation affecting the counts of neutrons registered by the sensor^19^. The slight impact of coniferous trees can be supported by the same equivalent layer thickness "seen" (28 cm) in all pixels within the Wigry site consisting of four pixels (S0, S1, and S4–S6) used mostly as arable lands and two pixels (S2 and S3) covered by coniferous trees. (Fig. 1) In connection with this, a recent study in the tropics on the Tibetan Plateau revealed^11^ that dense vegetation and associated small SMOS/SMAP-derived penetration depth need to be calibrated in contrast to limited or no vegetation in the dessert area exhibiting large penetration depth. El Hajj et al.^14^ reported that the L bands (in HH polarization) penetrated a well-developed canopy cover of wheat and grasslands at the Normalized Difference Vegetation Index (NDVI) > 0.7 (the backscattered L-HH is sensitive to soil moisture), whereas the penetration of the C-band into the canopy was limited for an NDVI < 0.7.

The variability in the equivalent layer thickness derived from the SMOS satellite among the pixels in the Białowieża site within Białowieża National Park can be further affected by the remaining dead trees. The presence of water accumulated in dead trees (not included in the estimation of soil moisture derived from the SMOS satellite) may have resulted in the lowest water depth "seen" (8 cm) in the soil in the forested areas within pixels S0 and S6 (Fig. 1) of the station. This effect can be more influenced by fallen than standing trees because the former can accumulate water^41^. It is worth noting that the low equivalent layer thickness "seen" in unforested pixel S2 can be influenced by organic matter in the marshes (52%) occurring in this pixel (Fig. 1). We observed visually that organic matter-rich soils (S1, S4) were saturated with water for most of the year and their topsoil is moist or sometimes dry only in the summer. In pixel S2 (in Belarus), the low "seen" calibrated layer thickness can also be influenced by the high content of silt (to 40%) and clay (to 11%) in the soil cover and organic matter in marshes.

The effect of vegetation on the "seen" calibrated layer thickness (*d*_*bias*_) is also visible while comparing the grassed (vegetated) and bare (arable) on the same (loess) soil within the Felin station*.* The greater calibrated layer thickness derived from the SMOS satellite for the grassed than bare soil (18 vs. 16 cm) can be a result of water uptake by grasses and the associated decrease in the soil water content and dielectric constant. On the other hand, in spite of the water uptake in soil covered by vegetation vs. bare soil, the dielectric constant can be higher in the former due to the lower evaporation from the soil surface^30^. This indicates that the "seen" calibrated layer thickness is affected by interactions between soil texture and vegetation.

It is worth noting that the equivalent layer thicknesses (ELT) for the Majdanek and Felin sites varying from about 5 to 25 cm during the study period were comparable to “sensing depth” from 8 cm in March to 27 cm in July observed in the SMOSREX (Surface Monitoring Of the Soil Reservoir EXperiment) microwave measurement campaign with similar soil^31,42^. Considering different sensing depths in the SMOSREX revealed that the agreement in soil moisture between the model-predicted data with consideration of the brightness temperature in the L band and measured data was satisfactory at depth of 0–5 cm which is concurrent with global surface soil moisture provided by the SMOS satellite^23^. In the case of spatially variable soils, the compliance of soil moisture from satellite and ground measurements in soil profile can be improved by using combined active, passive microwave and optical methods, with different penetration depths in airborne flights^43^. On the other hand, the obtained real and imaginary dielectric constants from the measurements of the SMOS brightness temperature allow to determine penetration depth and soil moisture using available methods^23,28,42^. The *SWEX_PD* and the approach proposed in this paper allowed for the determination of the calibrated and equivalent layer thicknesses which are consistent with the sensing depths (8–27 cm) obtained in the SMOSREX measurement campaign^42^ and justify the use of SMOS SM data in our study. More detailed investigations are needed to address these interactions and enhance the usability of the satellite *SM* products (retrievals).

## Summary and conclusions

The following findings were shown in this study:

1. The *SWEX_PD* was proposed for the first time to assess soil water resources based on 4-year SMOS satellite data and to determine the equivalent layer thickness derived from the SMOS satellite. The assessments were verified in nine sites under different soils and land cover (vegetation) in Poland.
2. The Bland–Altman plots including the *bias*, limits of agreements, and confidence intervals showed that the effect of a random factor was not significant and indicated that the soil water resources and equivalent layer thickness values were estimated with satisfactory accuracy.
3. Depending on the site conditions, the values of the equivalent layer thickness varied from 8.3 to 28.7 cm. This variation was mostly attributed to differences in soil texture, organic matter, and vegetation and interactions between them. It was observed that the negative effect of the sand content on equivalent layer thickness values was masked (or compensated for) by the abundant organic matter in marsh soils and broad-leaved forest cover. The higher values of the equivalent layer thickness for the grassed than bare silt (loess) soil were ascribed to plant water absorption in the former.
4. Highly heterogeneous conditions in terms of soil organic matter, textural composition, wetness, and vegetation in one site situated within Białowieża National Park were reflected in a wide range of equivalent layer thickness values (8.26–16.09 cm) and spatial variability of the soil water resources (CV < 98.8%).
5. Further studies are intended to address the impact of the soil conditions and land cover more comprehensively to improve the assessment of soil water resources using satellite retrievals.
